#  Lower Preoperative Verbal Memory Performance Is Associated with Delirium after Coronary Artery Bypass Graft Surgery: A Prospective Cohort Study

**DOI:** 10.1093/arclin/acac064

**Published:** 2022-08-01

**Authors:** Jakub Kaźmierski, Piotr Miler, Agnieszka Pawlak, Joanna Woźniak, Emilia Frankowska, Karina Nowakowska, Katarzyna Kuchta, Michał Pazdrak, Katarzyna Woźniak, Radosław Magierski, Michał Krejca, Mirosław Wilczyński

**Affiliations:** Department of Old Age Psychiatry and Psychotic Disorders, Medical University of Lodz, Lodz, Poland; Central Clinical Hospital, Medical University of Lodz, Lodz, Poland; Central Clinical Hospital, Medical University of Lodz, Lodz, Poland; Department of Old Age Psychiatry and Psychotic Disorders, Medical University of Lodz, Lodz, Poland; Department of Old Age Psychiatry and Psychotic Disorders, Medical University of Lodz, Lodz, Poland; Department of Old Age Psychiatry and Psychotic Disorders, Medical University of Lodz, Lodz, Poland; Department of Old Age Psychiatry and Psychotic Disorders, Medical University of Lodz, Lodz, Poland; Central Clinical Hospital, Medical University of Lodz, Lodz, Poland; Department of Cardiac Surgery, Central Clinical Hospital, Medical University of Lodz, Lodz, Poland; Department of Old Age Psychiatry and Psychotic Disorders, Medical University of Lodz, Lodz, Poland; Department of Cardiac Surgery, Central Clinical Hospital, Medical University of Lodz, Lodz, Poland; Department of Cardiac Surgery, Central Clinical Hospital, Medical University of Lodz, Lodz, Poland

**Keywords:** Delirium, Verbal memory, Cognitive impairment, Cardiovascular disease, Coronary artery bypass grafting

## Abstract

**Objective:**

Cognitive impairment constitutes one of the major risk factors of delirium after coronary artery bypass graft (CABG) surgery; however, it is unclear whether only patients with global cognitive decline are at increased risk for delirium or if individuals with preserved global cognitive functions but impairments in specific cognitive domains are also more vulnerable to developing delirium. Thus, this study aimed to analyze the neurocognitive status of patients scheduled for CABG surgery with the use of an advanced computerized cognitive battery (CNS Vital Signs) and to investigate possible associations between impaired performance in selective cognitive areas and the risk of postoperative delirium development.

**Methods:**

The study enrolled 127 participants with a median age of 67 years (IQR: 63–71). Postoperative delirium developed in 32 (25%) patients.

Before surgery, the patients were screened for global cognitive impairment with the use of the Mini-Mental State Examination Test, and the individuals were asked to perform the CNS Vital Signs battery to investigate 12 specific cognitive domains. The Confusion Assessment Method and the Memorial Delirium Assessment Scale were used to screen for a diagnosis of delirium postoperatively.

**Results:**

In multivariate models, a lower score of verbal memory-assessed preoperatively was independently associated with the risk of postoperative delirium development. Other independent predictors of delirium included more advanced age, gender female, depression, postoperative pyrexia, and the presence of extracorporeal circulation.

**Conclusions:**

As decreased verbal memory constitutes an independent risk factor for postoperative delirium, a verbal memory test may be a useful predictor of postoperative delirium development.

## Introduction

Postoperative delirium is a life-threatening syndrome encountered in the first 5 days following surgery. The incidence of postoperative delirium varies depending on patients’ population, surgical procedure, and diagnostic tools; however, it is most common after cardiac and orthopedic interventions (34–60%) (Kaźmierski et al., 2021; Rudolph & Marcantonio, 2011).

Patients with postoperative delirium experience poorer outcomes including an increased risk of death, compared with those who do not develop delirium (Park & Kim, 2019).

Furthermore, postoperative delirium including delirium after cardiac surgery is associated with prolonged hospitalization, perioperative complications, and a higher risk of discharge to nursing facilities (Kazmierski et al., 2014; Kazmierski, Banys, Latek, Bourke, & Jaszewski, 2013a).

Several well-established factors increase the risk of delirium after cardiac surgery. Those with the biggest impact include advanced age, compromised cognitive status, depression, presence of cardiopulmonary bypass (CPB), abnormal laboratory findings such as perioperative anemia, hypoxia, and inflammation (Greaves et al., 2020; Kazmierski et al., 2010a; Raats, van Eijsden, Crolla, Steyerberg, & van der Laan, 2015).

Cognitive decline constitutes one of the major risk factors of delirium after cardiac surgery (Greaves et al., 2020; Kazmierski et al., 2014). This association is observed particularly among study populations with a high prevalence of cognitive impairment such as individuals with vascular diseases, diabetes, or advanced age. Unfortunately, the biological basis of the relationship between cognitive impairment and delirium has not been elucidated. Furthermore, it is unclear whether only patients with global cognitive decline are at increased risk for delirium or if individuals with preserved global cognitive functions but impairments in specific cognitive domains are also more vulnerable to developing delirium.

Impairments in specific cognitive domains could be the explanation for the high vulnerability of nondemented elderly patients to postsurgery delirium (Bail et al., 2015). This study aimed to analyze the neurocognitive status of patients scheduled for coronary artery bypass graft surgery (CABG) with the use of CNS Vital Signs, a computerized cognitive battery,

and to investigate possible associations between impaired performance in selective cognitive areas and the risk of postoperative delirium development.

## Materials and Methods

We conducted a prospective cohort study at Central Clinical Hospital, Medical University of Lodz from April 2017 to December 2019.

The Bioethics Committee of the Medical Universty of Lodz reviewed and approved the study protocol. All participants provided written informed consent. Consecutive patients aged 18 years or older scheduled for elective CABG were eligible for participation in the study. The exclusion criteria were: acute intervention; unstable physical condition; and a diagnosis of global cognitive disorder. All recruited patients were White and Polish-speaking. Patients who underwent CABG were included both in the case of on-pump (without CPB) and off-pump (with CPB) surgery; however, the impact of CPB on the risk of postoperative delirium was controlled in the statistical analysis. Prior to scheduled surgery, we used the Polish version of a paper-and-pencil test (the Mini-Mental State Examination; MMSE) to assess patients’ cognitive status (Folstein, Folstein, & McHugh, 1975). Participants were screened for global cognitive disorder and were excluded if they scored <27 on the MMSE.

Together with the above cognitive screening test, the patients were asked to perform a computerized cognitive test battery: CNS Vital Signs (CNS VS) to investigate 12 specific cognitive domains (Gualteri & Johnson, 2006). Preoperative assessments were conducted in the hospital ward within the 3 days before surgery.

CNS VS is comprised of seven neuropsychological measures, including a verbal memory test (VBM), visual memory test (VIM), finger tapping test (FTT), symbol digit coding, a Stroop test (ST), a shifting attention test, and a continuous performance test. The battery used in the study calculated 12 clinical neurocognitive domains scores: Neurocognitive Index, Composite Memory, Verbal Memory, Visual memory, Psychomotor Speed, Reaction Time, Complex Attention, Cognitive Flexibility, Processing Speed, Executive Function, Simple Attention, and Motor Speed. The CNS VS test is administered on a Windows-based PC and takes about 30 min. The system grades severity of impairment based on an age-matched normative comparison database and the scores may be presented as percentiles. CNS VS standardized scores have a mean of 100 and a standard deviation is 15. CNS VS has a respectable normative database and it is sensitive to the most common causes of cognitive impairment (1069 subjects aged 7–90 participated in the normative database for CNS VS). Test–retest reliability was evaluated in 99 individuals, and the results were comparable to those achieved by equivalent conventional and computerized tests (Gualtieri & Johnson, 2006). Also, concurrent validity analysis conducted in 180 subjects indicates correlations that are comparable to the concurrent validity of similar tests. Discriminant validity of CNS VS is supported by studies of patients with mild cognitive impairment and dementia, postconcussion syndrome, severe traumatic brain injury, ADHD, and depression (Gualteri & Johnson, 2006).

An ongoing episode of depression and the presence of anxiety disorders (generalized anxiety disorder, panic disorder, mixed anxiety disorder) were evaluated by study psychiatrists during a clinical interview on the basis of DSM-5 criteria (American Psychiatric Association, 2013). The presence of the current episode of depression and current anxiety disorders was entered into statistical analysis.

Delirium was diagnosed with the use of the Confusion Assessment Method for the Intensive Care Unit (CAM-ICU) and Memorial Delirium Assessment Scale (MDAS) with the cut-off score of 10 (Ely et al., 2001; Kazmierski et al., 2008). Individuals were assessed once a day within the first 5 days after surgery. The data related to delirium screening were stored in the study file on the hospital ward until the screening was completed. All evaluations were performed by a study psychiatrist. If there was an inconsistency between the diagnostic tools regarding the delirium diagnosis, the final consensus was established within the study team physicians collecting information from all available sources. We decided to use two different scales for delirium screening since the studies show that the incidence of postoperative delirium varies depending on the tools used. The CAM-ICU and DSM criteria for delirium are reported to be more sensitive than MDAS and ICD-10 (Kazmierski et al., 2010b). Therefore, we believe that the use of two scales that are based on two different diagnostic systems increased the accuracy of the diagnostic process in the current study.

### Anesthesia and surgery

Anesthesia included the following procedures: induction: fentanyl 5–10mcg/kg, propofol 1–2.5 mg/kg and rocuronium 0.6–1.0 mg/kg; maintenance: fentanyl in continuous intravenous infusion in doses of 2–10mcg/kg/h, propofol 3–10 mg/kg/h and interrupted doses of rocuronium. Ventilation was provided with a breathing mixture of FiO2 0.5 and air to maintain end-tidal CO2 at 35–45 mmHg. From surgical incision to CPB connection sevoflurane 0.5–2 vol% was used. After surgery, patients stayed in the ICU on mechanical ventilation and were sedated with morphine in a continuous infusion of 1–2 mg per hour and propofol perfusion at a rate of 1–2 mg/kg/h. Patients who underwent CABG were operated through median sternotomy on CPB under normothermia. The anterograde DelNido cardioplegia was used for cardiac protection during the operation. In some cases, patients who underwent coronary revascularization were operated without CPB (CABG-OPCAB), on a beating heart, either through the median sternotomy or through left-sided mini-thoracotomy.

### Statistical analysis

Quantitative variables are expressed as medians and interquartile ranges (IQRs). For categorical variables, the number of observations (*n*) and a fraction (%) were calculated.

Normality was tested using Shapiro–Wilk’s test for normality. Differences between two independent samples for continuous data were analyzed using the Mann–Whitney U test (since the distributions of variables were different from normal). The effect size for continuous variables was measured with the rank-biserial correlation coefficient.

For categorical variables, statistical analysis was based on the chi-squared test or Fisher’s exact test. Cramer’s V coefficient was calculated to assess the effect size for categorical variables. Spearman’s rank correlation coefficients were calculated to assess the correlation between two quantitative variables. Nonparametric analysis of variance of aligned rank transformed data (ART) was used to compare the CNS VS scores in different groups of patients (taking into account two qualitative factors). Partial eta-squared was calculated as the effect size measure. The minimum study sample size was calculated using the power analysis, estimating the expected effects from our previous studies and assuming an alpha level of 0.10 and a power of 80% (the minimum sample size for each group is 37 patients). Initially, baseline and perioperative variables were evaluated for univariate association with postoperative delirium. Factors significant in univariate comparisons (*p* < .10) were included in a forward stepwise logistic regression model to identify the set of the independent risk factors for delirium. The results were considered significant for *p* < .05. All of the calculations were performed using STATISTICA (version 13.3, 2017; StatSoft, Inc., Tulsa, OK, USA).

## Results

During the study period, 163 patients were scheduled for CABG surgery and were assessed with regard to the study participation criteria. Of these, 15 patients were excluded due to the following reasons: 7 individuals had a diagnosis of cognitive impairment and 8 patients refused to participate. Among 148 participants who signed informed consent and were enrolled, 21 patients had an incomplete or invalid cognitive assessment. Therefore, finally, we enrolled into the analysis 127 individuals with a median age of 67 years (IQR: 63–71; 24 [18.9%] women). Postoperative delirium was diagnosed in 32 (25.2%) participants. Demography, baseline comorbidity, and cognitive performance characteristics of the whole studied population are presented in Tables 1 and 2. The univariate and multivariate associations between perioperative variables and delirium are shown in Tables 3–5.

**Table 1:** Demography, comorbidities, and perioperative characteristics of the study participants^*^

**Demography and baseline comorbidities**	*n*	IQR/%
Age (years)	67	(63–71)
Gender male	103	81.10%
Years of education	12	10–13
Depression	21	16.5%
Anxiety disorders	9	7.0%
Alcohol addiction	10	7.9%
MMSE Score	28	(26–30)
COPD	8	(6.30%)
Anemia	21	16.5%
Urea concentration (mmol/l)	6.7	(5.5–7.7)
Creatinine concentration (μmol/l)	86	(74–99)
Peripheral vascular disease	16	12.6%
Atrial fibrillation	22	12%
Arterial hypertension	104	82%
Diabetes	44	35%
Cerebrovascular disease	14	11.0%
**Perioperative characteristics**		
Surgery duration (h)	4	(3–4.5)
Presence of ECC	88	69.3%
Aortic cross-clamping time (min.)	40	(31–55)
Post-op pCO2 ≥ 45 (mmHg)	31	24.41%
Pot-op pO2 ≤ 60 (mmHg)	24	18.90%
Post-op hyperthermia	14	11%

COPD: Chronic Obstructive Pulmonary Disease; ECC: Extracorporeal Circulatory Support; MMSE: Mini-Mental State Examination.

^*^For continuous variables, the medians and interquartile ranges (IQRs) are given; for categorical variables, the number of observations (*n*) and a fraction (%) were calculated

**Table 2:** Distribution of normal and abnormal CNS VS scoring among the study participants^*^

**Cognitive domain**	**Very low score *n* (%)**	**Low score *n* (%)**	**Low average score *n* (%)**	**Average score *n* (%)**	**Above average score *n* (%)**
Neurocognitive index	35 (27.5)	21 (16.5)	29 (23)	40 (31.5)	2 (1.5)
Complex memory	18 (14)	30 (23.5)	29 (23)	43 (34)	7 (5.5)
Verbal memory	29 (23)	20 (15.7)	22 (17.3)	42 (33)	14 (11)
Visual memory	11 (8.7)	19 (15)	35 (27.5)	44 (34.6)	18 (14.2)
Processing speed	21 (16.5)	31 (24.4)	31 (24.4)	32 (25.2)	12 (9.5)
Executive functions	30 (23.6)	20 (15.7)	19 (15)	47 (37)	11 (8.7)
Psychomotor speed	27 (21.2)	24 (18.8)	28 (22)	33 (26)	15 (12)
Motor speed	22 (17.3)	10 (8)	20 (15.7)	54 (42.5)	21 (16.5)
Reaction time	42 (33)	25 (20)	23 (18)	30 (23.5)	7 (5.5)
Simple attention	33 (26)	19 (15)	24 (18.9)	50 (38.5)	2 (1.6)
Complex attention	45 (35.4)	10 (8)	22 (17.3)	43 (33.8)	7 (5.5)
Cognitive flexibility	35 (27.6)	22 (17.3)	15 (11.8)	44 (34.6)	11 (8.7)

^*^The classification of the scores achieved by the patients is based on the following CNS VS grading: Above average > 109 (standard score), >74 (percentile score); Average: 90–109, 25–74; Low average: 80–89, 9–24; Low: 70–79, 2–8; Very low: <70, <2.

**Table 3:** Demography and baseline neuropsychiatric characteristics analyzed in univariate analysis^*^

**Variable**	**Non-delirious** ^**a**^ **(*n* = 95)**	**Delirious** ^**a**^ **(*n* = 32)**	**Effect size** ^**b**^	***p*** ^**c**^
Age (years)	66 (61–70)	70 (67–74)	−0.353	**<.001**
Gender female	13 (13.7%)	11 (34.4%)	0.229	**<.01**
Depression	7 (7.4%)	14 (43.7%)	0.425	**<.001**
Anxiety disorders	4 (4.2%)	5 (15.6%)	0.193	**.044**
Alcohol addiction	7 (7.4%)	3 (9.38%)	0.032	.712
MMSE score	28 (26–30)	28 (27–30)	0.026	.669
Neurocognitive Index score	16 (2–40)	3 (1–15)	0.300	**<.010**
Complex memory score	88 (82–93)	82.5 (74.5–90)	0.273	**<.021**
Verbal memory score	46 (43–51)	43.5 (37.5–47)	0.284	**.016**
Visual memory score	41 (37–44)	40 (37–42)	0.165	.164
Processing speed score	32 (25–41)	26.5 (20–33.5)	0.332	**.005**
Executive functions	22 (1–38)	5.5 (−7 to 27.5)	0.256	**.030**
Psychomotor speed score	133 (118–148)	110.5 (94–131)	0.401	**<.001**
Motor speed score	101 (89–112)	87.5 (64.5–96)	0.375	**.001**
Reaction time (sec.)	868 (721–1030)	923 (868.5–1067.5)	−0.216	.068
Simple attention score	38 (36–39)	38 (35.5–38)	0.155	.186
Complex attention score	18 (1–31)	26.5 (20–31,5)	0.216	.068
Cognitive flexibility score	21 (−5 to 37)	0.5 (−14 to 22.5)	0.923	**.013**

MMSE: Mini-Mental State Examination.

^a^For continuous variables, the medians and interquartile ranges (IQRs) are given; for categorical variables, the number of observations (n) and a fraction (%) were calculated.

^b^For continuous variables rank-biserial correlation coefficient was calculated; for categorical variables, Cramer’s V coefficient was given.

^c^Significant associations are in bold.

^*^The raw patients’ scores are presented.

**Table 4:** Physical comorbidity and perioperative characteristics analyzed in univariate analysis

**Variable**	**Non-delirious** ^**a**^ **(*n* = 116)**	**Delirious** ^**a**^ **(*n* = 61)**	**Effect size** ^**b**^	***p*** ^**c**^
Peripheral vascular disease	8 (8.4%)	8 (25.0%)	0.217	**.027**
Diabetes	29 (30.5%)	15 (46.8%)	0.149	**.093**
Arterial hypertension	76 (80.0%)	28 (87.5%)	0.085	.341
NYHA	2 (2–2)	2 (2–3)	−0.081	.398
Atrial fibrillation^d^	8 (8.4%)	3 (9.4%)	0.015	1.000
Urea concentration (mmol/l)^d^	6.7 (5.6–7.7)	6.4 (5.3–7.7)	0.092	.558
Creatinine concentration (mcmol/l)^d^	84.8 (75.8–98.8)	88.5 (70.0–98.50)	−0.016	.949
Anaemia^d^	13 (13.6%)	8 (15.0%)	0.132	1.000
Cerebrovascular disease^d^	10 (10.5%)	4 (12.5%)	0.027	.750
COPD	5 (5.3%)	3 (9.4%)	0.073	.415
CCS	2 (2–3)	2 (2–3)	−0.072	.440
ECC	62 (65.3%)	26 (81.25%)	0.150	**.090**
Hyperthermia^e^	7 (7.4%)	7 (21.9%)	0.044	.201
Aortic cross-clamping (min.)	40 (31–55)	41 (28–55)	0.066	.783
Duration of surgery (h)	4 (3–4.5)	3.9 (3.4–4.3)	−0.046	.704
pCO2≥45^e^ (mmHg)	21 (22.1%)	10 (31.2%)	0.092	.298
pO2≤60^e^ (mmHg)	15 (15.8%)	9 (28.1%)	0.137	.123

CCS: Canadian Cardiovascular Society Degree; COPD: Chronic Obstructive Pulmonary Disease; ECC: Extracorporeal Circulation; NYHA: New York Heart Association grade.

^a^For continuous variables, the medians and interquartile ranges (IQRs) are given; for categorical variables, the number of observations (*n*) and a fraction (%) were calculated.

^b^For continuous variables, rank-biserial correlation coefficient was calculated; for categorical variables, Cramer’s V coefficient was given.

^c^Significant associations are in bold.

^d^Preoperative variables.

^e^Postoperative variables.

**Table 5:** Factors independently associated with delirium after cardiac surgery revealed in multivariate stepwise logistic regression analysis^*^

Variable	Coefficient	Standard Error	OR (95%CI)	*p*
Depression^a^	3.50	0.79	33.08 (7.08–154.56)	<.001
Age	0.15	0.06	1.16 (1.06–1.27)	<.001
Gender female	2.15	0.73	8.58 (2.04–36.19)	.003
Hyperthermia^b^	2.57	0.87	13.06 (2.36–71.95)	.003
Verbal memory^a^	−0.10	0.05	0.90 (0.82–0.99)	.033
Extracorporeal circulation	1.46	0.73	4.30 (1.03–17.87)	.045
Constant	−9.35	3.93	−	.017

^*^The regression model is statistically significant: χ2 = 26.162, df = 6, *P* < 0.001; Hosmer–Lemeshow test: χ2 = 4.566, *P* = 0.802; Nagelkerke *R*^2^ = 0.503.

^a^Preoperative variables.

^b^Postoperative variable.

In univariate analysis, only a decrease in complex attention, simple attention, reaction time, and visual memory tests was not associated with the higher risk of postoperative delirium. Patients with lower scores on tests evaluating verbal memory, complex memory, processing speed, executive functions, psychomotor speed, motor speed, cognitive flexibility, and neurocognitive index were more likely to develop delirium. However, in multivariate models, only decreased verbal memory assessed preoperatively was independently associated with the risk of postoperative delirium development. Other independent predictors of delirium included more advanced age, gender female, MDD, postoperative pyrexia, and the presence of CPB (Table 5). According to receiver operating characteristic analysis, the most optimal cutoff values of verbal memory score that predict the development of delirium were ≤40 with a sensitivity of 40.6% and a specificity of 83.2%, a positive predictive value of 44.8%, and a negative predictive value of 80.6% (odds ratio = 3.38; 95%CI: 1.39–8.20; area under the curve = 0.642; 95% confidence interval = 0.526–0.758; standard error = 0.059).

The possible relationships between impaired cognitive domains and the presence of psychiatric comorbidities (depression, anxiety disorders) and physical comorbidities (diabetes, hypertension, atrial fibrillation) were investigated. The ART ANOVA analysis revealed a statistically significant interaction between diabetes and delirium concerning the psychomotor speed scores (partial eta-squared = 0.05) and motor speed scores (partial eta-squared = 0.04). According to the post hoc pairwise comparisons, median preoperative psychomotor speed was decreased (longer time of tasks performance) among patients with a preoperative diagnosis of diabetes who developed postoperative delirium when compared with patients with delirium, though, without diabetes (118; IQR: 103–143 vs. 102; IQR: 86–122; *p* = .013). Also, median preoperative motor speed was decreased among patients with a preoperative diagnosis of diabetes who developed postoperative delirium when compared with patients with delirium and without diabetes (93; IQR: 83–98 vs. 81; IQR: 52–88; *p* = .011). Furthermore, a significant negative correlation between preoperative urea concentration and processing speed among individuals who experienced delirium was observed (Spearman’s rank correlation coefficients −0.431; *p* < .014). There were no significant interactions between other cognitive domains and comorbidities.

## Discussion

The study investigated the association between cardiovascular disease (CVD) patients’ performance in specific cognitive domains assessed with the use of the CNS VS battery and delirium after cardiac surgery. In univariate analysis, lower scores on tests evaluating verbal memory, complex memory, processing speed, executive functions, psychomotor speed, motor speed, cognitive flexibility, and neurocognitive index were associated with postoperative delirium development. However, in the multivariate logistic regression model, only decreased verbal memory independently predisposed to delirium development.

Verbal memory and verbal fluency decline are frequently the first-line symptoms of dementia due to Alzheimer’s disease (ad; Allison et al., 2021). Magnetic resonance imaging studies indicate positive associations between verbal memory measures and bilateral hippocampal volumes among patients with MCI and probable ad (Bonner-Jackson, Mahmoud, Miller, & Banks, 2015). According to other studies, the levels of ad biomarkers are related to the verbal memory scores achieved by the patients. For instance, in a biomarkers study, Reijs et al. (2017) revealed that decreased cerebrospinal fluid (CSF) Aβ42 and increased CSF t-tau correlate most strongly with a decline in scores of wordlist delayed recall test and wordlist learning when compared with other memory tests (Reijs et al., 2017). Judging from the above studies, we can conclude that verbal memory decline may constitute the marker of ad pathology. Based on the current findings, it can be also hypothesized that individuals with impaired verbal memory without global cognitive decline are more prone to delirium similarly to patients with symptomatic dementia due to ad or mixed dementia types (Fong, Davis, Growdon, Albuquerque, & Inouye, 2015).

CVD risk factors such as hypertension, diabetes, hyperlipidemia, cigarette smoking, obesity, and lack of physical activity substantially increase the risk of vascular cognitive impairment and dementia (Gorelick et al., 2011). However, these traditional cardiovascular risk factors were also recognized as increasing the risk of ad development (Barnes & Yaffe 2011) and likely contribute to mixed dementia cases that are often seen in community-dwelling cohorts (Gorelick, Counts, & Nyenhuis, 2016; Schneider, Arvanitakis, Bang, & Bennett, 2007).

Summing up, it is possible that in the current study conducted among CABG patients, domain-specific impairment signifies the underlying neuropathology. In this concept, delirium is unmasking this pathology, which in the age range and specificity of the studied population could be ad or mixed dementia. The participants included in the present study experienced cognitive abnormalities which are characteristic for ad (verbal memory decline), as well as vascular pathology (impaired executive functions). Poor test scores for executive functions were frequent in the studied population; however, lower verbal memory performance had a significant impact on the risk of postoperative delirium (Tables 2 and 5).

In previous studies, the associations between impaired executive functions measured with the Trail Making Test Part B, and postoperative delirium were found (Kazmierski, Banys, Latek, Bourke, & Jaszewski, 2013b; Rudolph et al., 2006). Other domain-specific cognitive impairments were not evaluated which may explain the inconsistency between the results of the aforementioned studies and the present findings.

It should be noted, however, that cognitive scores below average were frequent among the current population with the highest number of poor results recorded for domains reflecting executive functions—reaction time and cognitive flexibility (low and very low scores counted together were present in 53% and 45% of patients, respectively; Table 2). On the other hand, the lowest frequency of poor scores was recorded for visual memory and motor speed (23.7% and 25.3%, respectively; Table 2).

The studies systematically investigating the cognitive functioning of CVD patients are scarce; however, their results are similar to those of our analysis. In Gayda et al.’s (2017) study, coronary heart disease patients had significantly worse performance in tests related to working memory, processing speed, and cognitive flexibility compared with age-matched and young healthy controls. In addition, the decline of long-term verbal memory was revealed. In another study, the change from baseline to 72 months among coronary artery disease patients compared with healthy controls was evaluated. There was a mild but significant decline in executive functions and visuoconstruction, and a late decline (from 12 to 72 months) in verbal memory (Selnes et al., 2009).

The previous studies pointed out the impact of diabetes on cerebrovascular disease and delirium (Chen, Mo, Hu, Ou, & Luo, 2021; Zhou, Zhang, & Lu, 2014). About 20%–40% of patients with type 2 diabetes suffer from cerebral blood vessel diseases which often coexist with CVD. Furthermore, diabetic cerebrovascular disease is the main cause of death in this group. The major clinical manifestations are asymptomatic cerebral atherosclerosis, stroke, or cerebral small vessel disease (Zhou et al., 2014). Interestingly, in the present study, there were differences in baseline psychomotor speed and motor speed scores among delirium patients depending on the presence of diabetes diagnosis. Delirium patients with a diagnosis of diabetes performed poorer in the above tests compared with delirium patients without diabetes; however, such a difference was not detected among nondelirium subjects. It is possible that patients who are more prone to delirium had additional burdens (more frequent depression and cognitive impairment) which together with diabetes-associated complications (small vessels and nerves damage) contributed to psychomotor and motor performance decline.

According to the available studies, cardiovascular patients are characterized with increased psychiatric comorbidity compared with the general population. They suffer more frequently from depressive episodes, anxiety, and cognitive impairment (de Bruijn et al., 2015; Deckers et al., 2017; Kazmierski et al., 2010a, 2013a). Both major depressive disorder and compromised cognitive status were described as predictors of delirium after cardiac surgery (Itagaki et al., 2020; Kazmierski et al., 2010a, 2013a). In the present study, 44% of delirium patients had an ongoing episode of depression diagnosed before surgery compared with 7.4% of patients without postoperative delirium. Furthermore, the depressive episode was a significant predictor of postoperative delirium development (*p* < .001).

Advanced age is another risk factor for postsurgery delirium; however, the biological basis and underlying mechanisms of this association are poorly understood (Kazmierski et al., 2010a; Kaźmierski et al., 2021). It can be hypothesized that elderly patients with preserved global cognitive status and functioning suffer from subtle deficits in specific cognitive areas. The current study showed that elderly CABG subjects without global cognitive impairment experience an isolated decline in one or more cognitive domains, which in turn increases the risk of postoperative delirium.

The strengths of the present study include its prospective design, in-depth and detailed analysis of the cognitive status of participants, and multifactorial analysis of potential perioperative delirium risk factors.

The limitations of the present study should be also noted. Unfortunately, our analysis did not include such potential risk factors of delirium as multiple medications use, medications with a high cholinergic index, and the doses of anesthetics used. Furthermore, a diagnosis of global cognitive impairment (an exclusion criterion) was established on the basis of MMSE screening without the use of more sophisticated dementia diagnostic tools (CSF ad biomarkers or imaging ad biomarkers). On the other hand, dementia was not the primary condition investigated in the current study, and the whole diagnostic process was conducted by study psychiatrists which should limit the risk of false diagnosis.

In conclusion, impairment of specific cognitive domains is frequent among patients with CVD. As decreased verbal memory constitutes an independent risk factor for postoperative delirium, a verbal memory test may be a useful predictor of postoperative delirium development.
